# Table tennis for patients with Parkinson’s disease: A single-center, prospective pilot study

**DOI:** 10.1016/j.prdoa.2020.100086

**Published:** 2020-12-30

**Authors:** Kenichi Inoue, Shinsuke Fujioka, Koichi Nagaki, Midori Suenaga, Kazuki Kimura, Yukiko Yonekura, Yoshiki Yamaguchi, Kosuke Kitano, Ritsuko Imamura, Yoshinari Uehara, Hitoshi Kikuchi, Yoichi Matsunaga, Yoshio Tsuboi

**Affiliations:** a Department of Neurology, Murakami Karindoh Hospital, Fukuoka, Japan; b Department of Neurology, Fukuoka University School of Medicine, Fukuoka, Japan; c Department of Pharmaceutical Science, Tokushima Bunri University, Tokushima, Japan; d Murakami Karindoh Hospital, Fukuoka, Japan; e Faculty of Sports and Health Science, Fukuoka University, Fukuoka, Japan

**Keywords:** Parkinson’s disease, Table tennis, Motor symptoms, Non-motor symptoms, Exercise, Activities of daily living, safety

## Abstract

**Introduction:**

Table tennis is a popular sport worldwide. However, no study has examined whether it is an effective exercise for patients with Parkinson’s disease (PD). The efficacy and safety of table tennis exercise for PD patients was examined.

**Methods:**

This 6-month prospective study investigated if our table tennis exercise program could improve parkinsonian motor symptoms, cognition and psychiatric symptoms. Twelve PD patients with Hoehn & Yahr stage ≤4 were recruited. Patients participated in a 6-hour exercise session once weekly. All patients were assessed with the Movement Disorder Society Unified Parkinson’s Disease Rating Scale (MDS-UPDRS) parts I-IV, Montreal Cognitive Assessment (MoCA), Frontal Assessment Battery (FAB), Self-Rating Depression Scale (SDS), and Starkstein Apathy Scale (SAS) at baseline, 3 months, and 6 months.

**Results:**

Nine of 12 PD patients were analyzed, except for three patients for which data was missing. MDS-UPDRS parts II and III were improved at 3 months (median −4.0, p = 0.012 and median −10.0, p = 0.012) and 6 months (median −7.0, p = 0.015 and median −12.0, p = 0.008), whereas MDS-UPDRS total parts I scores and total IV scores, MoCA, FAB, SDS, and SAS were unchanged. Adverse events included fall and backache in one patient each.

**Conclusion:**

A table tennis exercise program is relatively safe and may improve activities of daily living and motor symptoms in patients with PD.

## Highlights

• This study reports the impact of table tennis exercises on Parkinson's disease.
• Table tennis exercise may improve motor symptoms of Parkinson's disease.
• Table tennis exercise may improve ADL of patients with Parkinson's disease.
• Table tennis exercise is safe for Parkinson's disease patients.

## 1 Introduction

Parkinson's disease (PD) is a progressive neurodegenerative disorder for which neither disease-modifying therapy nor curative therapy is available [1], [2], [3]. In addition to medications and device-aided therapies, such as deep brain stimulation (DBS) and levodopa carbidopa intestinal gel (LCIG) therapy, rehabilitation is strongly recommended to help patients maintain activities of daily living (ADL) [1], [2], [3]. Rehabilitation therapy is a treatment that is relatively simple and can be performed regardless of location, depending on the severity of individual patients, without causing serious complications. Some evidence supports the effects of exercise with regards to physical functioning, health-related quality of life, strength, balance, and gait speed for patients with PD [4], [5], [6]. Various physical interventions have also been introduced, including tai chi [7], [8], [9], [10], robot-assisted walking training [11], Lee Silverman Voice Treatment (LSVT®) BIG [12], music therapy [13], boxing [14], dance exercises [15], and exercise using video games [16]. Exercise is the only option for possible neuro-protection, presumably by increasing mitochondrial energy, stimulating antioxidant activity, reducing inflammation, causing angiogenesis, and producing synaptogenesis [17].

Table tennis is a popular exercise worldwide and can be enjoyed by individuals regardless of age or sex. Some groups of patients with PD have already started activities incorporating table tennis exercises [18]. Playing table tennis potentially requires agility, rapidity, and visual acuity to respond to the ball and partner, compared to other exercises that have been proven effective against PD. In addition, table tennis may be advantageous over physical interventions in that it is an activity that patients can enjoy as a game by competing for points. These potential strengths may further improve the outcome for PD patients. However, to the best of our knowledge, the efficacy and safety of a table tennis exercise program for patients with PD have not been investigated. Therefore, a prospective pilot study was conducted to determine if a table tennis exercise program could improve motor and non-motor symptoms of patients with PD, as well as to estimate the effect size and sample size.

## 2 Methods

### 2.1 Participants

Consecutive patients were recruited, and those who provided their informed consent were included in the study. Twelve outpatients from Murakami Karindoh Hospital with Hoehn & Yahr stage ≤ 4 PD were included in this study. They were diagnosed as having PD based on the International Parkinson and Movement Disorder Society (MDS) clinical diagnostic criteria for PD. The Hoehn & Yahr stage was assessed during “on” periods of PD. Patients with dementia scores ≤ 24 on the Mini-Mental State Examination [19], moderate and severe psychiatric disorders, musculoskeletal problems, parkinsonism other than PD, malignant tumors, and sequelae of neurological disorders other than PD were excluded. PD patients who were not independent in their daily lives during on-time were also excluded from this study.

### 2.2 Program

A 6-month prospective study was conducted from November 2018 to May 2019 to test if our table tennis exercise program could improve motor and non-motor symptoms in patients with PD. All participants were right-handed and participated in a 6-hour exercise session once a week for 6 months. Patients were not prohibited from adding new physical activity during the study.

Instructions regarding table tennis were provided by students from the Faculty of Sports and Health Science at Fukuoka University during the exercise. Before starting table tennis practice, participants stretched their bodies for 30 min (preparatory exercises). The practice included rally-style and game-style play, and participants alternated between each style. Medical staffs behind each patient watched closely so that they could immediately support them if they were likely to fall during play. Details of the program are available in Table 1.

**Table 1:** Schedule of the table tennis exercise program for Parkinson’s disease patients.

9:00 a.m.–9:30 a.m.	Assessment of vital signs and physical examination by a medical doctor
9:30 a.m.–10:00 a.m. Preparatory exercise	• Deep breath: 5 times • Neck bending (lateral, forward, backward) and neck rotation: 2 sets • Elbow flexion and extension: 10 times • Arm flexion and extension: 10 times • Wrist shaking: 10 times • Foot stepping: 20 times • Knee flexion and extension: 10 times • Horizontal foot stepping: 10 times • Ankle flexion and extension: 10 times • Achilles tendon extension: 10 times • Squat: 10 times • Deep breath: 5 times
10:00 a.m.–a0:30p.m. Table tennis exercise (practice)	• Practice swinging: forehand, backhand, forehand and backhand: 20 times • Rally practice: forehand or backhand drive, forehand and backhand drive, free drive • Medical staff stay behind patients just in case
10:30p.m.–1:30p.m. Lunch time	
1:30p.m.–2:00p.m. Preparatory exercise	• Deep breath: 5 times • Neck bending (lateral, forward, backward) and neck rotation: 2 sets • Elbow flexion and extension: 10 times • Arm flexion and extension: 10 times • Wrist shaking: 10 times • Foot stepping: 20 times • Knee flexion and extension: 10 times • Horizontal foot stepping: 10 times • Ankle flexion and extension: 10 times • Achilles tendon extension: 10 times • Squat: 10 times • Deep breath: 5 times
2:00p.m.–2:45p.m. Table tennis exercise (game & rally)	Game • Patients vs. patients or patients vs. medical staff • Referee: medical staff • Serve is changed every two times (in case of deuce, serve is changed every time) Rally • Patients vs. patients or patients vs. medical staff • Players substitution every 5 min
2:50p.m.–3:00p.m. Concluding exercises	• Stretch • Self-assessment of fatigue and fun by each patient

### 2.3 Assessments

All patients were assessed with the Movement Disorder Society Unified Parkinson’s Disease Rating Scale (MDS-UPDRS) parts I-IV at baseline, 3 months, and 6 months by a certified movement disorder specialist (S.F.). Cognitive and psychiatric assessments including the Montreal Cognitive Assessment (MoCA) [20], Frontal Assessment Battery (FAB) [21], Self-Rating Depression Scale (SDS) [22], and Starkstein Apathy Scale (SAS) [23] were done by an experienced speech therapist (K.K.). MDS-UPDRS was assessed during “on” periods of PD. Anti-parkinsonian drugs could be added as needed based on the patients’ symptoms. Adverse events such as falls, injuries, and pain were also assessed. The Institutional Review Board (IRB) of Murakami Karindoh Hospital approved this study, and all participants provided their written, informed consent. This study was not registered in a clinical study registry. Patient’s backgrounds and clinical features at baseline are available in Table 2.

**Table 2:** Patient’s backgrounds and clinical features at baseline.

N = 9 (men = 2/women = 7)	Mean (SD)
Age (year)	71.8 (7.2)
mean disease duration (year)	7.5 (4.3)
Hoehn-Yahr	3.0 (0.4)
Mini-Mental State Examination, MMSE	28.8 (1.5)
Montreal Cognitive Assessment, MoCA	24.9 (3.2)
Frontal Assessment Battery, FAB	14.9 (1.5)
Self-Rating Depression Scale, SDS	45.7 (10.3)
Starkstein Apathy Scale, SAS	16.3 (6.9)

### 2.4 Statistical analysis

All analyses were performed using the statistical software package IBM SPSS statistics V.26 (SPSS Japan, Tokyo, Japan). Values are reported as means ± SD or medians and interquartile ranges (25%, 75%). The analysis was performed on patients measured for all periods. Friedman's test was used to compare the three groups (baseline, 3 months, and 6 months), and P value <0.05 was considered significant. The post hoc test was then conducted using Bonferroni-type multiple comparisons analysis for items that were significantly different, and P value <0.016 was considered significant. Since this study was a comparison that was based on a non-parametric test, the effect size (ES) *r* was used. The test statistic was set back to Z and calculated as *r* = Z / √N. The effect size is small for 0.1, medium for 0.3, and large for 0.5 and higher [24].

## 3 Results

**Fig. 1:**
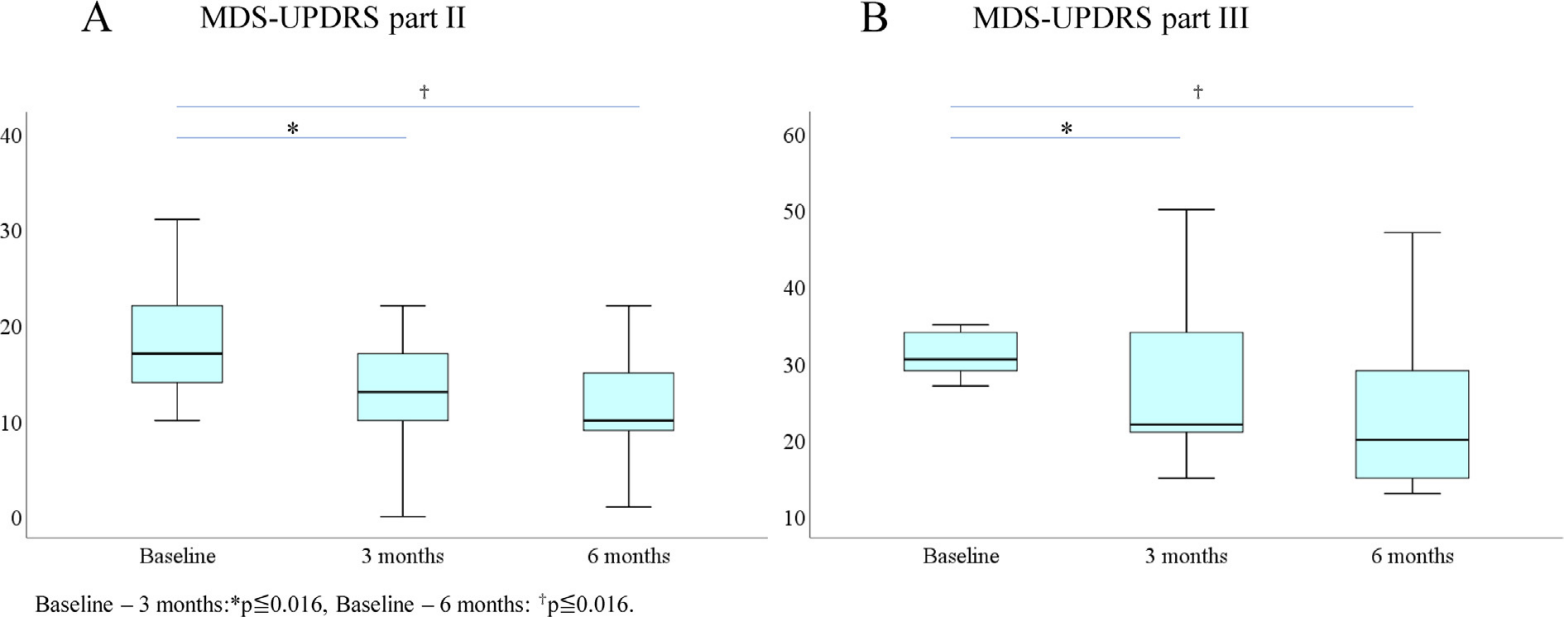
Changes in median scores of the MDS-UPDRS part II and part III. MDS-UPDRS, Movement Disorder Society Unified Parkinson’s Disease Rating Scale.

All patients completed the program throughout the 6 months. However, only 9 (7 women, 2 men) of the 12 patients completed measurements for the entire period (baseline, 3 months, 6 months), so the 3 patients were excluded from analysis. The mean age was 71.8 ± 7.2 years, and the mean disease duration was 7.5 ± 4.3 years. The mean Hoehn & Yahr stage was 3.0 ± 0.4. Six patients showed wearing off, and one had dyskinesia at baseline. Eight patients were treated with oral medications, and the mean levodopa equivalent dose was 500.0 ± 175.3 mg/day. One participant was treated with DBS without any oral anti-parkinsonian drugs. Some patients had fallen several times before enrollment in the study, but all of them walked without using auxiliary devices such as canes and walkers during “on” periods. Some patients used push carts during “off” periods and when they went out. All patients performed physical activities before starting the study, and none of them began additional new physical activities during the 6-month study. Bonferroni’s-type multiple comparison analysis showed that MDS-UPDRS parts II (motor experiences of daily living) and III (motor examination) were improved at 3 months (median −4.0, p = 0.012 and median −10.0, p = 0.012, respectively) and 6 months (median −7.0, p = 0.015 and median −12.0, p = 0.008) (Fig. 1, Table 3), whereas MDS-UPDRS total part I scores (non-motor experiences of daily living) and total IV scores (motor complications) were unchanged (Table 3). MoCA, FAB, SDS, and SAS were unchanged. Bonferroni’s-type multiple comparison analysis for MDS-UPDRS part II showed the subscores of speech (baseline – 3 months, p = 0.013) and getting out of bed, a car, or a deep chair (baseline – 6 months, p = 0.013) were improved. Neck of rigidity assessed by MDS-UPDRS part III was improved at both 3 and 6 months from baseline (baseline – 3 months, p = 0.002 and baseline – 6 months, p = 0.003) (Supplemental Table). Adverse events included fall and backache in one patient each. Fortunately, one who fell was not injured, and the other was mild enough to not affect continuous exercise. No participant dropped out during the six months. Effect sizes were: MDS-UPDRS total score 0.81 (baseline – 3 months), 0.81 (baseline – 6 months); MDS-UPDRS part II 0.84 (baseline – 3 months) and 0.84 (baseline – 6 months); and MDS-UPDRS part III 0.81 (baseline – 3 months) and 0.89 (baseline – 6 months). During the study, no additional anti-parkinsonian drugs were added to any of the patients’ therapeutic regimens. Regarding the MDS-UPDRS part I and IV, the total scores and each subscore were not significantly different between baseline and 6 months.

**Table 3:** Comparison of 3 groups for MDS-UPDRS scores.

MDS-UPDRS		Baseline		3 months		6 months		*p*-value
Total Scores	Mean (SD)	67.4	(15.9)	53.3	(15.3)	50.8	(18.8)	0.013
	Median (IQR)	73.0	(26)	53.0*	(21.5)	56.0†	(36.5)	
Part Ⅰ Scores	Mean (SD)	10.8	(4.8)	10.8	5.7	12.4	(7.0)	0.105
	Median (IQR)	12.0	(3.5)	11.0	(8.0)	11.0	(12.0)	
Part Ⅱ Scores	Mean (SD)	18.3	(6.8)	13.0	(6.2)	11.6	(6.0)	0.002
	Median (IQR)	17.0	(10.5)	13.0*	(7.0)	10.0†	(8.0)	
Part III Scores	Mean (SD)	35.0	(9.4)	26.9	(10.8)	24.7	(11.8)	0.002
	Median (IQR)	32.0	(11.5)	22.0*	(14)	20.0†	(18.5)	
Part Ⅳ Scores	Mean (SD)	3.3	(3.8)	2.7	(3.0)	2.1	(2.7)	0.348
	Median (IQR)	2.0	(6.5)	2.0	(6.0)	0.0	(4.5)	

*p*-value is Freadman’s test.

Bonferroni’s-type multiple comparison analysis.

Baseline – 3 months:*p ≦ 0.016, Baseline – 6 months: †*p* ≦ 0.016.

## 4 Discussion

A prospective pilot study was conducted to determine if a table tennis exercise program could improve motor and non-motor symptoms of PD patients. The first of the key results of the study was that table tennis exercise significantly improved MDS-UPDRS parts II and III. The present research provides evidence that a table tennis exercise program can be safe and effective at improving some aspects of motor function seen in daily life and motor symptoms of patients with PD with a Hoehn & Yahr stage ≤ 4. Because swinging paddles repeatedly around the body requires manipulation of axial muscles [3], [25], it is possible that this exercise program may help ameliorate axial symptoms. Furthermore, the rhythmic sounds of the ball hitting the table may provide an auditory cue for participants to move [26], [27]. In addition, the visual image of an orange or white ping-pong ball coming over a green table may provide an exciting visual cue for participants to move [28]. It was also noted that table tennis rehabilitation improved motor experiences of daily living and motor symptoms not only at 6 months, but also even at 3 months. The results indicate that the rehabilitation using table tennis may have relatively immediate positive effects on PD patients.

It can be inferred that the effect size of our table tennis exercise program is comparable or greater than that of regular physical therapy [29]. Our table tennis exercise program is different from LSVT®-BIG in that the latter is an exercise in which patients repeatedly perform a specific formal exercise. Previous reports have confirmed that LSVT®-BIG therapy effectively improves motor symptoms in PD patients. In the Berlin LSVT®-BIG Study that randomly compared LSVT®-BIG, Nordic walking, and domestic unsupervised exercises, patients who underwent LSVT®-BIG improved their UPDRS motor score by a mean of 5.05 points during 4-month follow-up [30]. In another study, patients who underwent LSVT®-BIG for 6 months improved their UPDRS motor scores by a mean of 6.8 points [31]. On the other hand, the table tennis exercise program, performed once per week for 6 months, had a positive impact on UPDRS part III scores of 12.0 points, which comparable to LSVT®-BIG exercise.

The second of the key results of the study was that the table tennis exercise program could be performed by patients with moderate to advanced PD without major adverse events during the 6 months. The mean Hoehn & Yahr stage of the participants was 3.0 ± 0.4, and the participants included patients with motor complications such as wearing off and dyskinesia, and a patient who underwent DBS. This study may be highly appreciated because these patients were able to safely carry out the program without serious injury nor adverse events. In a randomized control trial to evaluate the effect of an exercise program for PD, 12.5% of participants developed joint pain (shoulder, back, and hip) [32]. Another study comparing effects of Tango dance with that of controls reported that 13% of controls and 22% of tango developed adverse events [33]. The percentage of patients who had adverse events of our study were similar to that of the studies. If well prepared, such as staffing for fall prevention, as in this study protocol, table tennis can be a relatively safe and well-tolerated activity for PD patients. Various kinds of exercises are available for patients with PD, although some require specific instructions. For example, tai chi is a traditional exercise in Asian countries and has been shown to be effective as rehabilitation therapy for PD patients [34], [35]. However, beginners need guidance and learn how to practice by themselves before starting a tai chi program. LSVT®-BIG therapy incorporates a program of aggressive trunk and limb functional motions and should only be administered by an LSVT®-BIG-certified therapist [31], [36], [37]. In addition, it is necessary to continue the program four times a week every week, which may be difficult for some patients. Table tennis is a familiar sport worldwide that can be enjoyed anywhere using a table, paddle, and ping-pong ball.

There are several other key points that can help ensure that patients can keep performing the activity for a long time without dropouts, including enough space and equipment, as well as staff to instruct them, and one of the most important tips is to maintain the patients’ motivation. Table tennis has elements of competition that other major rehabilitation exercises for PD do not have. Playing as a “match” can produce a positive effect in terms of reward processing. Competition can provide goals for patients. The goals urge patients to concentrate on the practice and also provide them with enjoyment. A final match was conducted to evaluate the results of table tennis practice for each participant on the final date of the program.

The third of the key results of this study is that cognitive or psychiatric symptoms were unchanged. However, the improvement in motor symptoms, but not in non-motor symptoms, may support the interpretation that the improvement in motor symptoms was not due to a placebo effect. For example, pain and depression are symptoms in which placebo treatment is most effective in patients with PD [38]. Therefore a placebo effect is more likely to improve both motor and non-motor symptoms, especially depression and other mental symptoms. Longitudinal and comparative studies are needed to confirm the efficacy of this program.

This study has several limitations. First, a single neurologist assessed motor function. Some potential bias could have been removed if we had been able to videotape the clinical evaluations and have a third person assess motor function without knowing when the videotapes were recorded during the study period. Second, this study had a small number of participants of a single ethnic group. Third, there was a predominance of female participants. Fourth, this study had no comparisons with controls and could not rule out the Hawthorne effect or a placebo effect. Fifth, it is not possible to determine if the improvements seen were related to possible reconditioning, the socialization aspects of the activity, or to the exercise itself, such as stretching or aerobics.

In conclusion, a table tennis exercise program is potentially safe and useful to improve ADL and motor symptoms of patients with PD, though future researches using control subjects are warranted to confirm the finding. It is also more convenient and easier to learn than other rehabilitation therapies available for PD. We are conducting a prospective, multi-center, randomized clinical study comparing the effectiveness of table tennis exercises for patients with PD with that of other rehabilitation therapies, including LSVT® and conventional rehabilitation therapy, such as stretching and exercise, in an attempt to isolate the effects of each exercise activity. Another question to address is, to get the most effectiveness from the exercise, whether an individual needs to compete with others or whether the same benefit could be seen merely by playing against a wall. Thus, a comparative study that evaluates the effects between interpersonal and individual playing is needed.

## Funding sources

This research did not receive any specific grant from funding agencies in the public, commercial, or not-for-profit sectors.

## Conflict of interest

This research did not receive any specific grant from funding agencies in the public, commercial, or not-for-profit sectors.

## Contributors

K. Inoue designed the study and evaluated the patients’ assessment throughout the study and wrote the draft of the manuscript. S. Fujioka assessed patients’ motor symptoms at baseline, 3 months, and 6 months. K. Nagaki and M. Suenaga analyzed the data, and revised the manuscript. K. Kimura performed a psychological test to assess non-motor symptoms. Y. Yonekura and Y. Yamaguchi instructed the patients in playing table tennis and watched carefully so that the patients did not fall or get injured. K. Kitano created an exercise schedule. R. Imamura and Y. Uehara dispatched table tennis instructors. H. Kikuchi and Y. Matsunaga helped refine the manuscript. Y. Tsuboi was involved in the planning and guidance of the written manuscript. All authors have read and approved the final version of the manuscript.
