# Supplementary material for: Table tennis for patients with Parkinson’s disease: A single-center, prospective pilot study
Source: Clin Park Relat Disord. 2020 Dec 30;4:100086. doi: 10.1016/j.prdoa.2020.100086 (PMC8299968; doi:10.1016/j.prdoa.2020.100086)
Supplement: Supplementary data 1 [file mmc1.docx]

Supplemental Table. Comparison of 3 groups for MDS-UPDRS part II and III subscores

|  |  | Baseline | 3 months | 6 months | *p-value* |
| --- | --- | --- | --- | --- | --- |
| No. | MDS-UPDRS part II, III  subscores | Mean (SD) | Mean (SD) | Mean (SD) |  |
| 2.1 | Speech | 1.6 (0.7) | 0.7 (0.5) | 0.8 (0.7) ^♱^ | 0.005 |
| 2.11 | Getting out of bed, car or deep chair | 1.8 (0.7) | 1.2 (0.7) * | 1.0 (0.9) | 0.008 |
| 3.3 | Rigidity, neck | 2.7 (0.7) | 1.7 (0.7) ** | 1.7 (1.0) ^♱♱^ | <0.001 |
| *p*-value is Freadman’s test.  Bonferroni’s-type multiple comparison analysis.  Baseline – 3 months:*p≦0.016, **p≦0.003, Baseline – 6 months: ^♱^p≦0.016, ^♱♱^p≦0.003. | | | | | |
